# Implication in finite posets with pseudocomplemented sections

**DOI:** 10.1007/s00500-022-07052-5

**Published:** 2022-04-19

**Authors:** Ivan Chajda, Helmut Länger

**Affiliations:** 1 Faculty of Science Department of Algebra and Geometry, Palacký University Olomouc, 17. listopadu 12, Olomouc, 771 46 Czech Republic; 2 Faculty of Mathematics and Geoinformation, Institute of Discrete Mathematics and Geometry, Wiedner Hauptstraße 8-10, Vienna, 1040 Austria

**Keywords:** Poset, Section, Relative pseudocomplement, Poset with pseudocomplemented sections, Intuitionistic implication, Unsharp implication, Unsharp conjunction, Unsharp residuation

## Abstract

It is well-known that relatively pseudocomplemented lattices can serve as an algebraic semantics of intuitionistic logic. To extend the concept of relative pseudocomplementation to non-distributive lattices, the first author introduced so-called sectionally pseudocomplemented lattices, i.e. lattices with top element 1 where for every element *y* the interval [*y*, 1], the so called section, is pseudocomplemented. We extend this concept to posets with top element. Our goal is to show that such a poset can be considered as an algebraic semantics for a certain kind of more general intuitionistic logic provided an implication is introduced as shown in the paper. We prove some properties of such an implication. This implication is “unsharp” in the sense that the value for given entries need not be a unique element, but may be a subset of the poset in question. Using this implication we show that we can even recover the order of the original poset. Further, a new “unsharp” operator $\odot$ of conjunction can be introduced which is adjoint to “unsharp” implication and hence we obtain an “unsharp” residuated poset.

## Introduction

Relatively pseudocomplemented lattices, often called Heyting algebras (see, e.g. Iturrioz 1977 and Köhler 1980) or Brouwerian lattices (see, e.g. Köhler 1981), arise from intuitionistic logic and were first investigated by T. Skolem about 1920, see also Frink (1962) and Balbes (1973). For a detailed development see, e.g. Curry (1977). Within this context, the relative pseudocomplement $x*y$ of *x* with respect to *y* is usually considered as intuitionistic implication, see, e.g. Nemitz (1965) or Curry (1977).

Hence, in relatively pseudocomplemented lattices we define

$$\begin{aligned} x\rightarrow y:= x*y. \end{aligned}$$

**Fig. 1:**
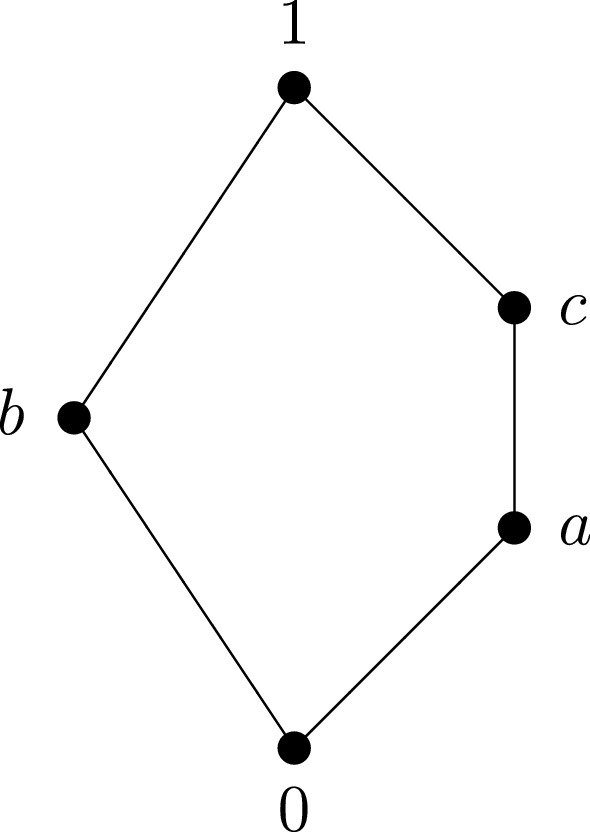
.

It is well-known that every finite pseudocomplemented lattice is distributive. This fact can be rather restrictive because not every algebraic semantics of a propositional logic forms a distributive lattice, e.g. the logic of quantum mechanics is not distributive. In order to extend investigations in intuitionistic logic also to the non-distributive case, the first author introduced so-called *sectionally pseudocomplemented lattices*, see Chajda (2003) and Chajda and Radeleczki (2003). If ${\mathbf {P}}=(P,\le ,1)$ is a poset with top element 1 and if $y\in P$ then we call the interval [*y*, 1] a section. There are lattices with top element 1 where for every element *y* and each $x\in [y,1]$ there exists a pseudocomplement $x^y$ of *x* with respect to *y*, see, e.g. Chajda (2003). Putting

1 $$\begin{aligned} x\rightarrow y:=(x\vee y)^y \end{aligned}$$

the situation becomes formally analogous to the case of relatively pseudocomplemented lattices. For the typical case, consider the lattice depicted in Fig. 1.

It is evident that this lattice has pseudocomplemented sections, but the lattice is neither relatively pseudocomplemented (since the relative pseudocomplement of *c* with respect to *a* does not exist) nor distributive.

The operation tables for $x^y$ and $\rightarrow$ look as follows:

$$\begin{aligned} \begin{array}{c|c@{\quad }c@{\quad }c@{\quad }c@{\quad }c} x^y &{} 0 &{} a &{} b &{} c &{} 1 \\ \hline 0 &{} 1 &{} - &{} - &{} - &{} - \\ a &{} b &{} 1 &{} - &{} - &{} - \\ b &{} c &{} - &{} 1 &{} - &{} - \\ c &{} b &{} a &{} - &{} 1 &{} - \\ 1 &{} 0 &{} a &{} b &{} c &{} 1 \end{array} \quad \quad \quad \begin{array}{c|c@{\quad }c@{\quad }c@{\quad }c@{\quad }c} \rightarrow &{} 0 &{} a &{} b &{} c &{} 1 \\ \hline 0 &{} 1 &{} 1 &{} 1 &{} 1 &{} 1 \\ a &{} c &{} 1 &{} b &{} 1 &{} 1 \\ b &{} c &{} a &{} 1 &{} c &{} 1 \\ c &{} b &{} a &{} b &{} 1 &{} 1 \\ 1 &{} 0 &{} a &{} b &{} c &{} 1. \end{array} \end{aligned}$$

The notion of relatively pseudocomplemented lattices was extended to posets, see, e.g. Chajda et al. (2020). It is useful when a reduct of intuitionistic logic is considered where one studies only the connective implication but not other connectives like disjunction or conjunction. Let us note that in intuitionistic logic, the connectives implication, conjunction and disjunction are independent.

## Posets with pseudocomplemented sections

In numerous cases when studying non-classical propositional logics, only the logical connective implication is considered. It means that we study only a reduct of the ordered structure where the implication is defined. Hence, the investigation of posets with pseudocomplementation in sections plays an important role. In order to extend our study also to (not necessarily relatively pseudocomplemented) posets with pseudocomplemented sections, let us introduce several necessary concepts.

Let $(P,\le )$ be a poset $a,b\in P$ and $A,B\subseteq P$. We say $A<B$ if $x\le y$ for all $x\in A$ and $y\in B$. Instead of $\{a\}<\{b\}$, $\{a\}<B$ and $A<\{b\}$ we simply write $a<b$, $a<B$ and $A<b$, respectively. Analogously we proceed with the relational symbols $\le$, > and $\ge$. Denote by

$$\begin{aligned} L(A):=\{x\in P\mid x\le A\}\text { and }U(A):=\{x\in P\mid A\le x\} \end{aligned}$$

the so-called *lower* and *upper cone* of *A*, respectively. Instead of $L(\{a\})$, $L(\{a,b\})$, $L(A\cup \{a\})$, $L(A\cup B)$ and $L\big (U(A)\big )$ we simply write *L*(*a*), *L*(*a*, *b*), *L*(*A*, *a*), *L*(*A*, *B*) and *LU*(*A*), respectively. Analogously, we proceed in similar cases. Denote the set of all minimal and maximal elements of *A* by ${{\,\mathrm{Min}\,}}A$ and ${{\,\mathrm{Max}\,}}A$, respectively.

Recall that a *pseudocomplemented poset* is an ordered quadruple $(P,\le ,{}^*,0)$ where $(P,\le ,0)$ is a poset with bottom element 0 and $^*$ is a unary operation on *P* such that for all $x\in P$, $x^*$ is the greatest element of $(P,\le )$ satisfying $L(x,x^*)=0$. (Here and in the following, we often identify singletons with their unique element.) This means that $x\wedge x^*$ exists for each $x\in P$ and $x\wedge x^*=0$. It is worth noticing that pseudocomplemented structures are studied recently by several authors, see, e.g. Chajda et al. (2020) and Venkatanarasimhan (1971) for pseudocomplemented posets, Nimbhorkar and Nehete (2021) and Talukder et al. (2021) for pseudocomplemented semilattices and Rao (2012) for pseudocomplemented distributive lattices.

Let us mention that in every logic, both classical or non-classical, a prominent role plays the logical connective implication. The reason is that implication enables logical deduction, i.e. the derivation of new propositions from given ones. In order to study a logic based on a poset, one cannot expect that the result of implication will be uniquely determined. This means that the result of the implication $x\rightarrow y$ for given elements *x* and *y* of a given poset *P* would be a subset of *P*, not necessarily a singleton. This is the reason why we will call such an implication “unsharp”. On the other hand, we ask such an unsharp implication to satisfy the rules and properties usually satisfied by an implication and, moreover, the results of our implication should be as high as possible. We introduce such an unsharp implication within the next section. In Proposition 2.4 we show that our implication satisfies properties similar to those satisfied by the standard implication. We also show that the values of results of our implication are usually higher than those for implication of intuitionistic logic based on relative pseudocomplementation. In the last section we introduce also an unsharp connective conjunction which is connected with our implication via a certain kind of adjointness.

It is worth noticing that implication in a propositional logic which is not distributive was already investigated, e.g. in Finch (1970), in bounded posets also in Zeman (1979) or in fuzzy posets in Ojeda-Hernández et al. (2022). Implication in sectionally pseudocomplemented posets was studied by J. Cīrulis in Cīrulis (2008) and a certain kind of adjointness was treated in Cornejo et al. (2021). Our method applied here is different. At first, we define the connective implication in an “unsharp” version contrary to the above mentioned papers. The essential advantage of our approach is that our implication is everywhere defined. Although the results of our unsharp implication are subsets, these contain only elements with the highest truth value. Hence, we consider also the case when there does not exists the greatest possible value of implication for given entries but several maximal values which are incomparable because of maximality.

### Definition 2.1

A *finite poset with pseudocomplemented sections* is an ordered quadruple $\big (P,\le ,(^y;y\in P),1\big )$ where $(P,\le ,1)$ is a finite poset with top element 1 and for every $y\in P$, $([y,1],\le ,{}^y,y)$ is a pseudocomplemented poset. For every $y\in P$ and every subset *B* of [*y*, 1] put $B^y:=\{b^y\mid b\in B\}$. Finally, for all $x,y\in P$ define the implication $x\rightarrow y$ as follows:

$$\begin{aligned} x\rightarrow y:=\big ({{\,\mathrm{Min}\,}}U(x,y)\big )^y. \end{aligned}$$

A *finite poset with 0 and pseudocomplemented sections* is an ordered quintuple $\big (P,\le ,(^y;y\in P),0,1\big )$ where $\big (P,\le ,(^y;y\in P),1\big )$ is a finite poset with pseudocomplemented sections and 0 is the bottom element of $(P,\le )$.

Observe that because of $1\in U(x,y)$ we have ${{\,\mathrm{Min}\,}} U(x,y)\ne \emptyset$.

### Remark 2.2

If $\big (P,\le ,(^y;y\in P),1\big )$ is a finite poset with pseudocomplemented sections, $b\in P$ and $a\in [b,1]$ then

$$\begin{aligned} a^b=\max \{x\in P\mid L(a,x)\cap [b,1]=b\}. \end{aligned}$$

Hence, in general, $\rightarrow$ is not a binary operation on *P* but an operator assigning to each element of $P^2$ a non-empty subset of *P*. The almost obvious relationship between the sectional pseudocomplementation and the operator $\rightarrow$ is as follows.

### Lemma 2.3

Let $\big (P,\le ,(^y;y\in P),1\big )$ be a finite poset with pseudocomplemented sections and $a,b\in P$. Then, the following hold:

(i) If $a\vee b$ exists in $(P,\le )$ then $a\rightarrow b=(a\vee b)^b$,
(ii) if $b\le a$ then $a\rightarrow b=a^b$.

### Proof

(i) If $a\vee b$ exists in $(P,\le )$ then
  $$\begin{aligned} a\rightarrow b= & {} \big ({{\,\mathrm{Min}\,}}U(a,b)\big )^b\\= & {} \big ({{\,\mathrm{Min}\,}}U(a\vee b)\big )^b=(a\vee b)^b. \end{aligned}$$
(ii) if $b\le a$ then because of (i) we have $a\rightarrow b=(a\vee b)^b=a^b$.

$$\square $$

In what follows we list some elementary but important properties of this implication. We can see that these are analogous to know properties of implication in classical and non-classical propositional calculus.

### Proposition 2.4

Let $\big (P,\le ,(^y;y\in P),1\big )$ be a finite poset with pseudocomplemented sections and $a,b\in P$. Then, the following hold:

(i) $a\le b$ if and only if $a\rightarrow b=1$,
(ii) if $a\vee b$ exists then $(a\vee b)\rightarrow b=a\rightarrow b$,
(iii) $$1\rightarrow a=a$$,
(iv) $$a\le b\rightarrow a$$,
(v) $$a\rightarrow (b\rightarrow a)=1$$.

### Proof

(i) The following are equivalent:
  $$\begin{aligned} a&\le b, \\ {{\,\mathrm{Min}\,}}U(a,b)&=b, \\ x&=b\text { for all }x\in {{\,\mathrm{Min}\,}}U(a,b), \\ x^b&=1\text { for all }x\in {{\,\mathrm{Min}\,}}U(a,b), \\ \big ({{\,\mathrm{Min}\,}}U(a,b)\big )^b&=1, \\ a\rightarrow b&=1, \end{aligned}$$
(ii) if $a\vee b$ exists then
  $$\begin{aligned} (a\vee b)\rightarrow b= & {} \big ({{\,\mathrm{Min}\,}}U(a\vee b,b)\big )^b\\ {}= & {} \big ({{\,\mathrm{Min}\,}}U(a\vee b)\big )^b\\ {}= & {} \big ({{\,\mathrm{Min}\,}}U(a,b)\big )^b=a\rightarrow b, \end{aligned}$$
(iii) $$\begin{aligned} 1\rightarrow a=\big ({{\,\mathrm{Min}\,}}U(1,a)\big )^a=\big ({{\,\mathrm{Min}\,}}U(1)\big )^a=1^a=a, \end{aligned}$$
(iv) $$a\le \big ({{\,\mathrm{Min}\,}}U(b,a)\big )^a=b\rightarrow a$$,
(v) this follows from (iii) and from (i) of Lemma 2.3.

$$\square $$

The next result shows that under appropriate assumptions our unsharp implication satisfies important properties already known from standard implication.

### Proposition 2.5

Let $\big (P,\le ,(^y;y\in P),1\big )$ be a finite poset with pseudocomplemented sections and $a,b,c\in P$. Then, the following hold:

(i) If $a\le b$ and $a\vee c$ exists in $(P,\le )$ then $b\rightarrow c\le a\rightarrow c$,
(ii) if $a\vee b$ exists in $(P,\le )$ then $a\le (a\rightarrow b)\rightarrow b$,
(iii) if $a\vee b$ exists in $(P,\le )$ then $a\rightarrow b=\big ((a\rightarrow b)\rightarrow b\big )\rightarrow b$.

### Proof

(i) Since $a\vee c$ exists in $(P,\le )$, we have $a\rightarrow c=(a\vee c)^c$ according to (i) of Lemma 2.3. Now, by (P2), everyone of the following assertions implies the next one:
  $$\begin{aligned} a&\le b, \\ U(b,c)&\subseteq U(a,c), \\ {{\,\mathrm{Min}\,}}U(b,c)&\subseteq U(a\vee c), \\ a\vee c&\le x\text { for all }x\in {{\,\mathrm{Min}\,}}U(b,c), \\ x^c&\le (a\vee c)^c\\&=a\rightarrow c\text { for all }x\in {{\,\mathrm{Min}\,}}U(b,c), \\ b\rightarrow c&=\big ({{\,\mathrm{Min}\,}}U(b,c)\big )^c\le a\rightarrow c \end{aligned}$$
(ii) Because of (P3) and (i) and (ii) of Lemma 2.3 we have
  $$\begin{aligned} a\le a\vee b\le \big ((a\vee b)^b\big )^b=(a\rightarrow b)\rightarrow b. \end{aligned}$$
(iii) Because of (i) of Lemma 2.3, (P4) and (ii) of Lemma 2.3 we have
  $$\begin{aligned} a\rightarrow b= & {} (a\vee b)^b=\Big (\big ((a\vee b)^b\big )^b\Big )^b\\ {}= & {} \big ((a\rightarrow b)\rightarrow b\big )\rightarrow b. \end{aligned}$$

$$\square $$

Let ${\mathbf {P}}=(P,\le )$ be a poset and $a,b\in P$. Recall the following definitions.

- The greatest element *x* of *P* satisfying $L(a,x)\subseteq L(b)$ is called the *relative pseudocomplement* $a*b$ of *a* with respect to *b*. The poset ${\mathbf {P}}$ is called *relatively pseudocomplemented* if any two elements of *P* have a relative pseudocomplement, see Chajda et al. (2020) and Venkatanarasimhan (1971).
- The greatest element *x* of *P* satisfying $L(U(a,b),x)=L(b)$ is called the *sectional pseudocomplement* $a\circ b$ of *a* with respect to *b*. The poset ${\mathbf {P}}$ is called *sectionally pseudocomplemented* if any two elements of *P* have a sectional pseudocomplement.

### Remark 2.6

Let $(P,\le ,1)$ be a poset with top element 1 and $a,b\in P$ with $b\le a$. Further assume that the sectional pseudocomplement $a\circ b$ of *a* with respect to *b* and the pseudocomplement of $a^b$ of *a* in [*b*, 1] exist. Then $a\circ b\le a^b$.

### Proof

Since $b\in L(b)=L\big (U(a,b),a\circ b\big )$, we have $b\le a\circ b$. Moreover,

$$\begin{aligned} L(a,a\circ b)\cap [b,1]= & {} L\big (U(a),a\circ b\big )\cap [b,1]\\ {}= & {} L\big (U(a,b),a\circ b\big )\cap [b,1]\\ {}= & {} L(b)\cap [b,1]=\{b\}. \end{aligned}$$

Hence $$a\circ b\le a^b$$. $$\square $$

**Fig. 2:**
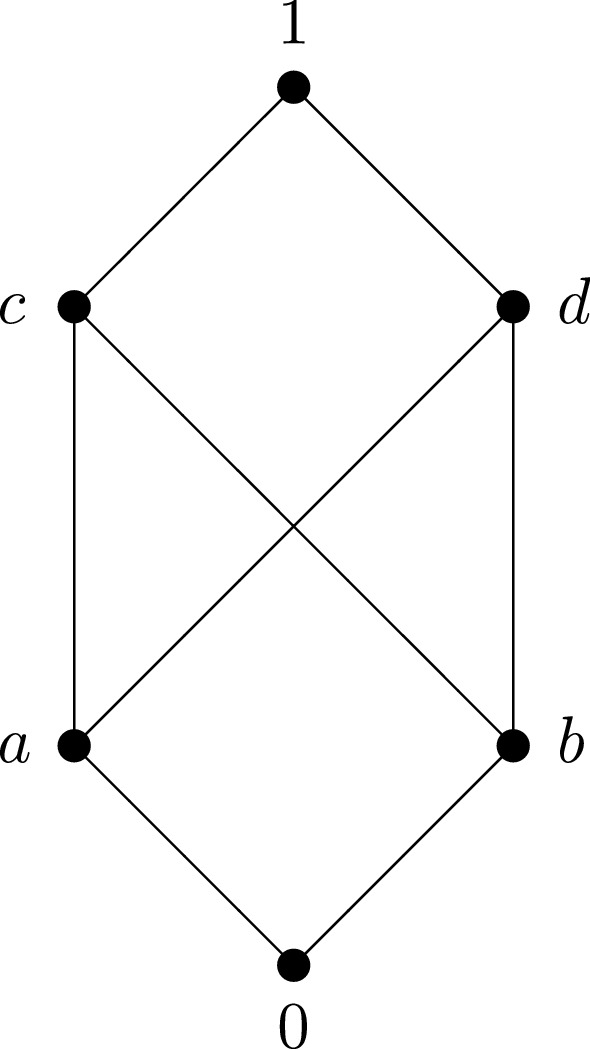
.

Let us note that the sectional pseudocomplement is not the same as the pseudocomplement in the corresponding section. For example, consider the poset depicted in Fig. 2. Then $a\notin [b,1]$. Thus, the pseudocomplement of *a* in the section [*b*, 1], i.e. $a^b$, does not exist. On the other hand, the sectional pseudocomplement $a\circ b$ of *a* with respect to *b* exists and is equal to *b* because *b* is the greatest element *x* satisfying $L\big (U(a,b),x\big )=L(b)$ since $U(a,b)=\{c,d,1\}$. It is worth noticing that $a\circ b$ differs from our unsharp implication $a\rightarrow b$ because $a\rightarrow b=\{c,d\}$.

### Example 2.7

The poset shown in Fig. 2.

has pseudocomplemented sections and is simultaneously relatively pseudocomplemented. The tables for $x^y$, $\rightarrow$ and $*$ look as follows:

$$\begin{aligned} \begin{array}{c|c@{\quad }c@{\quad }c@{\quad }c@{\quad }c@{\quad }c} x^y &{} 0 &{} a &{} b &{} c &{} d &{} 1 \\ \hline 0 &{} 1 &{} - &{} - &{} - &{} - &{} - \\ a &{} b &{} 1 &{} - &{} - &{} - &{} - \\ b &{} a &{} - &{} 1 &{} - &{} - &{} - \\ c &{} 0 &{} d &{} d &{} 1 &{} - &{} - \\ d &{} 0 &{} c &{} c &{} - &{} 1 &{} - \\ 1 &{} 0 &{} a &{} b &{} c &{} d &{} 1 \end{array} \quad \begin{array}{c|c@{\quad }c@{\quad }c@{\quad }c@{\quad }c@{\quad }c} \rightarrow &{} 0 &{} a &{} b &{} c &{} d &{} 1 \\ \hline 0 &{} 1 &{} 1 &{} 1 &{} 1 &{} 1 &{} 1 \\ a &{} b &{} 1 &{} \{c,d\} &{} 1 &{} 1 &{} 1 \\ b &{} a &{} \{c,d\} &{} 1 &{} 1 &{} 1 &{} 1 \\ c &{} 0 &{} d &{} d &{} 1 &{} d &{} 1 \\ d &{} 0 &{} c &{} c &{} c &{} 1 &{} 1 \\ 1 &{} 0 &{} a &{} b &{} c &{} d &{} 1 \end{array} \quad \begin{array}{c|c@{\quad }c@{\quad }c@{\quad }c@{\quad }c@{\quad }c} * &{} 0 &{} a &{} b &{} c &{} d &{} 1 \\ \hline 0 &{} 1 &{} 1 &{} 1 &{} 1 &{} 1 &{} 1 \\ a &{} b &{} 1 &{} b &{} 1 &{} 1 &{} 1 \\ b &{} a &{} a &{} 1 &{} 1 &{} 1 &{} 1 \\ c &{} 0 &{} a &{} b &{} 1 &{} d &{} 1 \\ d &{} 0 &{} a &{} b &{} c &{} 1 &{} 1 \\ 1 &{} 0 &{} a &{} b &{} c &{} d &{} 1. \end{array} \end{aligned}$$

The intuitionistic implication, i.e. the relative pseudocomplement $*$ differs from our “unsharp” implication $\rightarrow$, e.g. $a*b=b$ whereas $a\rightarrow b=\{c,d\}$. Hence, although $a\rightarrow b$ is an “unsharp” implication because its result is a two-element subset of *P*, its values *c* and *d* are greater than the value of intuitionistic implication $a*b$.

**Fig. 3:**
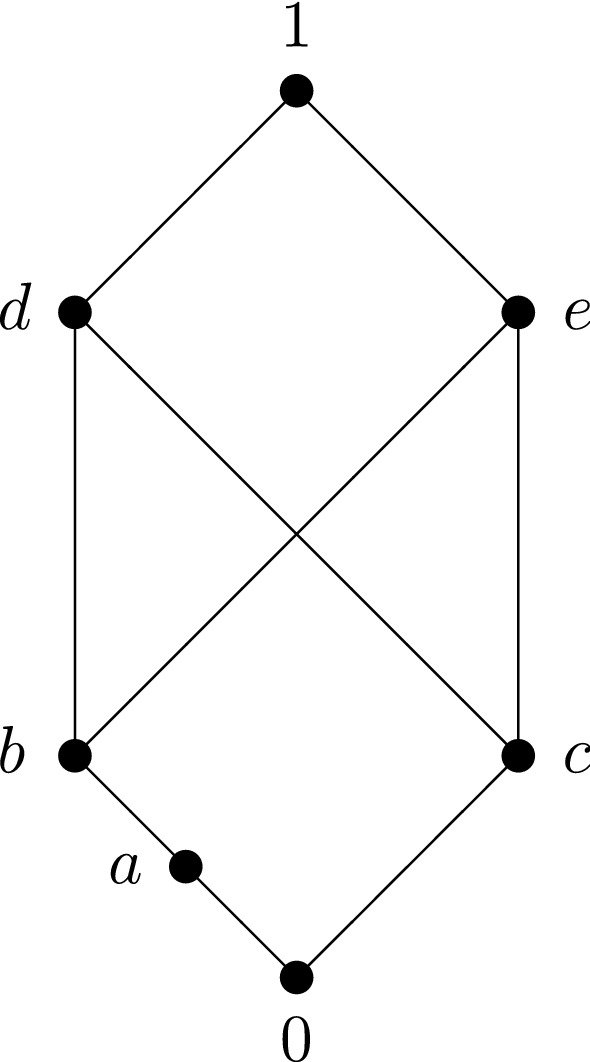
.

### Example 2.8

The poset shown in Fig. 3:

has pseudocomplemented sections, but is not relatively pseudocomplemented since the relative pseudocomplement of *b* with respect to *a* does not exist. The tables for $x^y$ and $\rightarrow$ look as follows:

$$\begin{aligned} \begin{array}{c|c@{\quad }c@{\quad }c@{\quad }c@{\quad }c@{\quad }c@{\quad }c} x^y &{} 0 &{} a &{} b &{} c &{} d &{} e &{} 1 \\ \hline 0 &{} 1 &{} - &{} - &{} - &{} - &{} - &{} - \\ a &{} c &{} 1 &{} - &{} - &{} - &{} - &{} - \\ b &{} c &{} a &{} 1 &{} - &{} - &{} - &{} - \\ c &{} b &{} - &{} - &{} 1 &{} - &{} - &{} - \\ d &{} 0 &{} a &{} e &{} e &{} 1 &{} - &{} - \\ e &{} 0 &{} a &{} d &{} d &{} - &{} 1 &{} - \\ 1 &{} 0 &{} a &{} b &{} c &{} d &{} e &{} 1. \end{array} \quad \quad \quad \begin{array}{c|c@{\quad }c@{\quad }c@{\quad }c@{\quad }c@{\quad }c@{\quad }c} \rightarrow &{} 0 &{} a &{} b &{} c &{} d &{} e &{} 1 \\ \hline 0 &{} 1 &{} 1 &{} 1 &{} 1 &{} 1 &{} 1 &{} 1 \\ a &{} c &{} 1 &{} 1 &{} \{d,e\} &{} 1 &{} 1 &{} 1 \\ b &{} c &{} a &{} 1 &{} \{d,e\} &{} 1 &{} 1 &{} 1 \\ c &{} b &{} a &{} \{d,e\} &{} 1 &{} 1 &{} 1 &{} 1 \\ d &{} 0 &{} a &{} e &{} e &{} 1 &{} e &{} 1 \\ e &{} 0 &{} a &{} d &{} d &{} d &{} 1 &{} 1 \\ 1 &{} 0 &{} a &{} b &{} c &{} d &{} e &{} 1. \end{array} \end{aligned}$$

It is a question if, having an operator $\rightarrow$ on a finite set *A*, it can be converted into a poset with pseudocomplemented sections. For this, we introduce the following structure.

## Implication algebras

Our next goal is to show that this unsharp implication in fact determines the given finite poset with pseudocomplemented sections. For this purpose we define the following concept.

### Definition 3.1

A *finite *I*-algebra* is an ordered triple $(A,\rightarrow ,1)$ with a finite set *A*, an operator $\rightarrow :A^2\rightarrow 2^A\setminus \{\emptyset \}$ and $1\in A$ satisfying the following conditions:

1. $$x\rightarrow x\approx x\rightarrow 1\approx 1$$,
2. $$x\rightarrow y=y\rightarrow x=1\Rightarrow x=y$$,
3. $$x\rightarrow y=y\rightarrow z=1\Rightarrow x\rightarrow z=1$$,
4. $$y\rightarrow z=z\rightarrow x=z\rightarrow (x\rightarrow y)=1\Rightarrow z=y$$,
5. $$\big (y\rightarrow x=y\rightarrow u=1\text { and }(y\rightarrow z=z\rightarrow x=z\rightarrow u=1\Rightarrow z=y)\big )\Rightarrow u\rightarrow (x\rightarrow y)=1$$,
6. $$x\rightarrow y=\{z\rightarrow y\mid x\rightarrow z=y\rightarrow z=1,\text { and }x\rightarrow u=y\rightarrow u=u\rightarrow z=1\Rightarrow u=z\}$$.

Now we can state and prove the following result.

### Theorem 3.2

Let ${\mathbf {P}}=\big (P,\le ,(^y;y\in P),1\big )$ be a finite poset with pseudocomplemented sections and put $x\rightarrow y:=\big (\min U(x,y)\big )^y$ for all $x,y\in P$. Then ${\mathbb {I}}({\mathbf {P}}):=(P,\rightarrow ,1)$ is a finite I-algebra.

### Proof

Let $a,b\in P$. According to (i) of Proposition 2.4, $a\le b$ if and only if $a\rightarrow b=1$, and according to (ii) of Lemma 2.3, $b\le a$ implies $a\rightarrow b=a^b$. Now (I1) follows since $\le$ is reflexive and 1 is the top element of $(P,\le )$, (I2) and (I3) follow by antisymmetry and transitivity of $\le$, respectively. Let $x,y,z,u\in P$. If $y\le z\le x$ and $z\le x^y$ then $z\in L(x,x^y)\cap [y,1]=\{y\}$, i.e. $z=y$ which shows that (I4) holds. Now for $x,u\in [y,1]$ the following are equivalent:

$$\begin{aligned} y\rightarrow z=z\rightarrow x=z\rightarrow u=1&\Rightarrow z=y, \\ z\in L(x,u)\cap [y,1]&\Rightarrow z=y, \\ L(x,u)\cap [y,1]&\subseteq \{y\}, \\ L(x,u)\cap [y,1]&=\{y\}. \end{aligned}$$

Since for $x,u\in [y,1]$, $L(x,u)\cap [y,1]=\{y\}$ implies $u\le x^y$, we have (I5). Finally, (I6) follows from the definition of $\rightarrow$. $\square$

However, also the converse of Theorem 3.2 is true, see the following result.

### Theorem 3.3

Let ${\mathbf {A}}=(A,\rightarrow ,1)$ be a finite I-algebra and define

$$\begin{aligned} x\le y&:\Leftrightarrow x\rightarrow y=1, \\ x^y&:=x\rightarrow y\text { whenever }y\le x \end{aligned}$$

($x,y\in A$). Then ${\mathbb {P}}({\mathbf {A}}):=\big (A,\le ,(^y;y\in A),1\big )$ is a finite poset with pseudocomplemented sections.

### Proof

Because of (I1) – (I3), $(A,\le ,1)$ is a finite poset with top element 1, because of (I4), $L(x,x^y)\cap [y,1]\subseteq \{y\}$ for all $x,y\in I$ with $y\le x$ and hence $L(x,x^y)\cap [y,1]=\{y\}$ for all $x,y\in I$ with $y\le x$, and because of (I5), $y\in A$, $x,u\in [y,1]$ and $L(x,u)\cap [y,1]=\{y\}$ imply $u\le x^y$. Hence for all $y\in A$, $([y,1],\le ,{}^y,y)$ is a pseudocomplemented poset. $\square$

### Remark 3.4

In the above proof, condition (I6) of Definition 3.1 is not needed. We need this condition in order to prove that the above described correspondence is one-to-one.

Now we show that the assignments from Theorems 3.2 and 3.3 are mutually inverse.

### Theorem 3.5

The correspondence described in Theorems 3.2 and 3.3 is one-to-one.

### Proof

Let ${\mathbf {P}}=\big (P,\le ,(^y;y\in P),1\big )$ be a finite poset with pseudocomplemented sections, put

$$\begin{aligned} {\mathbb {I}}({\mathbf {P}})&=(P,\rightarrow ,1), \\ {\mathbb {P}}\big ({\mathbb {I}}({\mathbf {P}})\big )&=\big (P,\le ',(_y;y\in P),1\big ) \end{aligned}$$

and let $a,b\in P$. Then because of the definition of $\le '$ and (i) of Proposition 2.4 the following are equivalent:

$$\begin{aligned} a\le & {} 'b, \\ a\rightarrow b= & {} 1, \\ a\le & {} b. \end{aligned}$$

If $b\le a$ then because of the definition of $a_b$ and (ii) of Lemma 2.3 we have $a_b=a\rightarrow b=a^b$ This shows ${\mathbb {P}}\big ({\mathbb {I}}({\mathbf {P}})\big )={\mathbf {P}}$. Now let ${\mathbf {A}}=(A,\rightarrow ,1)$ be a finite I-algebra, put

$$\begin{aligned} {\mathbb {P}}({\mathbf {A}})&=\big (A,\le ,(^y;y\in I),1\big ), \\ {\mathbb {I}}\big ({\mathbb {P}}({\mathbf {A}})\big )&=(A,\Rightarrow ,1) \end{aligned}$$

and let $a,b\in A$. Then

$$\begin{aligned} a\Rightarrow b= & {} \big ({{\,\mathrm{Min}\,}}U(a,b)\big )^b\\ {}= & {} \{x^b\mid a,b\le x,\text { and }a,b\le y\le x\text { implies }y=x\}\\ {}= & {} a\rightarrow b \end{aligned}$$

because of the definition of $\le$ and (I6). This shows ${\mathbb {I}}\big ({\mathbb {P}}({\mathbf {A}})\big )={\mathbf {A}}$. $\square$

In every finite poset $\big (P,\le ,(^y;y\in P),0,1\big )$ with 0 and pseudocomplemented sections one can define $\lnot x:=x\rightarrow 0$ for all $x\in P$. Observe that $\lnot x=\max \{y\in P\mid L(x,y)=0\}$ for all $x\in P$ and hence $\lnot x=x^0$ for all $x\in P$. Due to the fact that $\lnot x$ is the pseudocomplementation as defined usually (see, e.g. Balbes 1973 or Venkatanarasimhan 1971), it satisfies the known properties as follows:

1. $$\lnot 0=1$$ and $$\lnot 1=0$$,
2. $$x\le y$$ implies $$\lnot y\le \lnot x$$,
3. $$x\le \lnot \lnot x$$,
4. $$\lnot \lnot \lnot x=\lnot x$$.

### Remark 3.6

Condition (P2) expresses the fact that our negation and implication satisfy the contraposition law, i.e.

$$\begin{aligned} \text {if }x\rightarrow y=1\text { then also }\lnot y\rightarrow \lnot x=1. \end{aligned}$$

At the end of this section we show that every bounded pseudocomplemented poset contains a subposet where the unary negation $'$ is a complementation. This is in fact analogous to the Glivenko Theorem (see, e.g. Birkhoff 1979) for pseudocomplemented lattices.

### Proposition 3.7

Let ${\mathbf {P}}=(P,\le ,{}',0,1)$ be a bounded pseudocomplemented poset. Then $(P',\le ,{}',0,1)$ with $P':=\{x'\mid x\in P\}$ is a complemented poset.

### Proof

Clearly, $P'=\{x\in P\mid x''=x\}$. Let $a,b\in P'$. Then $a'\in P'$. Moreover, $L(a,a')=0$. If $b\in U(a,a')$ then $b'\in L(a',a'')=L(a,a')=0$ and hence $b=b''=0'=1$. This shows $U(a,a')=1$, i.e. $a'$ is a complement of *a*. $\square$

### Example 3.8

**Fig. 4:**
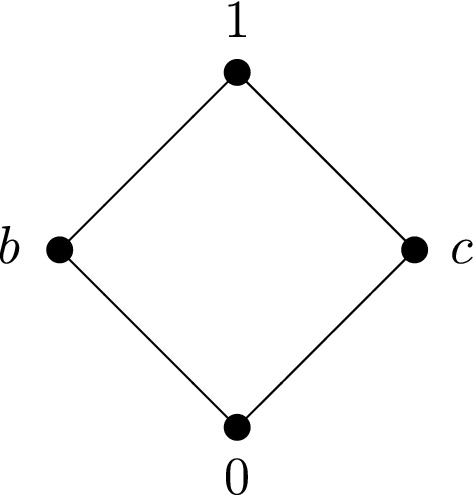
.

If $(P,\le ,{}',0,1)$ is the bounded pseudocomplemented poset of Example 2.8, then the complemented poset $(P',\le ,{}',0,1)$ is depicted in Fig. 4:

## Adjointness of implication with unsharp conjunction

It is known that every relatively pseudocomplemented lattice is residuated, in fact it is a “prototype” of a residuated lattice where the operation multiplication is considered as the lattice meet and the relative pseudocomplement as a residuum. As mentioned in the introduction, we define sectionally pseudocomplemented lattices in the sake to extend the concept of relative pseudocomplementation to non-distributive lattices. The question concerning residuation in sectionally pseudocomplemented lattices was answered by the authors and J. Kühr (2020) as follows.

A lattice ${\mathbf {L}}=(L,\vee ,\wedge ,\odot ,\rightarrow ,1)$ with top element 1 and with two binary operations $\odot$ and $\rightarrow$ is called *relatively residuated* if

(i) $(L,\odot ,1)$ is a commutative groupoid with 1,
(ii) $$x\le y$$ implies $$x\odot z\le y\odot z$$,
(iii) $(x\vee z)\odot (y\vee z)\le z$ if and only if $x\vee z\le y\rightarrow z$.

It is worth noticing that the class of relatively residuated lattices forms a variety, see Chajda et al. (2020). Namely, under condition (i), conditions (ii) and (iii) are equivalent to the identities

(iv) $$x\odot z\le (x\vee y)\odot z$$,
(v) $$z\vee y\le x\rightarrow \Big (\big ((x\vee y)\odot (z\vee y)\big )\vee y\Big )$$,
(vi) $$(x\rightarrow y)\odot (x\vee y)\le y$$.

Unfortunately, we cannot adopt this definition for posets $(P,\le )$ because we cannot use the lattice operations and, moreover, our implication is not an operation but an operator, i.e. its result need not be a singleton. However, we can proceed as follows. Having in mind that $\rightarrow$ is unsharp, we can introduce an unsharp connective conjunction as follows:

$$\begin{aligned} x\odot y:={{\,\mathrm{Max}\,}}L(x,y) \end{aligned}$$

and for non-singleton subsets *A*, *B* of *P* we define $A\odot B:={{\,\mathrm{Max}\,}}L(A,B)$. One can mention that this conjunction reaches the maximal possible values for given entries *x* and *y*. Moreover, the operator $\odot$ is idempotent since for every $x\in P$ we have

$$\begin{aligned} x\odot x={{\,\mathrm{Max}\,}}L(x,x)={{\,\mathrm{Max}\,}}L(x)=x. \end{aligned}$$

Now we can define the following concept.

### Definition 4.1

A poset ${\mathbf {P}}=(P,\le ,\odot ,\rightarrow ,1)$ with top element 1 and two operators $\odot$ and $\rightarrow$, both mappings from $P^2$ to $2^P$, such that

(i) $\odot$ is commutative and associative and $x\odot 1\approx x$,
(ii) if $x\le y$ and $z\in P$ then there exists some $t\in y\odot z$ with $x\odot z\le t$,
(iii) $z\in x\odot y$ if and only if $(z\le x,y$ and $x\le y\rightarrow z)$

will be called *unsharply residuated*. Condition (iii) will be called *unsharp adjointness*. We call an unsharply residuated poset ${\mathbf {P}}$ *divisible* if for all $x,y\in P$ with $x\ge y$ we have that $x\rightarrow y$ is a singleton and $\big (x\odot (x\rightarrow y)\big )\cap [y,1]=\{y\}$.

We are going to show that finite posets with pseudocomplemented sections are unsharply residuated and divisible.

### Theorem 4.2

Let $\big (P,\le ,(^y;y\in P),1\big )$ be a finite poset with pseudocomplemented sections and for $x,y\in P$ define

$$\begin{aligned} x\odot y&:={{\,\mathrm{Max}\,}}L(x,y), \\ x\rightarrow y&:=\big ({{\,\mathrm{Min}\,}}U(x,y)\big )^y. \end{aligned}$$

Then $(P,\le ,\odot ,\rightarrow ,1)$ is unsharply residuated and divisible.

### Proof

Let $$a,b,c\in P$$. Then

$$\begin{aligned} a\odot 1={{\,\mathrm{Max}\,}}L(a,1)={{\,\mathrm{Max}\,}}L(a)=a \end{aligned}$$

and, clearly, $\odot$ is commutative. Moreover,

$$\begin{aligned} (a\odot b)\odot c&={{\,\mathrm{Max}\,}}L\big ({{\,\mathrm{Max}\,}}L(a,b),c\big )\\&={{\,\mathrm{Max}\,}}\Big (L\big ({{\,\mathrm{Max}\,}}L(a,b)\big )\cap L(c)\Big ) \\&={{\,\mathrm{Max}\,}}\big (L(a,b)\cap L(c)\big )={{\,\mathrm{Max}\,}}L(a,b,c)\\&={{\,\mathrm{Max}\,}}\big (L(a)\cap L(b,c)\big ) \\&={{\,\mathrm{Max}\,}}\Big (L(a)\cap L\big ({{\,\mathrm{Max}\,}}L(b,c)\big )\Big )\\&={{\,\mathrm{Max}\,}}L\big (a,{{\,\mathrm{Max}\,}}L(b,c)\big )=a\odot (b\odot c). \end{aligned}$$

Thus $\odot$ satisfies (i) of Definition 4.1. If $a\le b$ then

$$\begin{aligned} a\circ c={{\,\mathrm{Max}\,}}L(a,c)\subseteq L(a,c)\subseteq L(b,c) \end{aligned}$$

and hence there exists some $d\in {{\,\mathrm{Max}\,}}L(b,c)$ with $a\odot c\le d$. This shows (ii) of Definition 4.1. Now unsharp adjointness remains to be proved. Because of Lemma 2.3 (ii) the following are equivalent:

$$\begin{aligned}&c\in a\odot b, \\&c\in {{\,\mathrm{Max}\,}}L(a,b), \\&L(a,b)\cap [c,1]=\{c\}, \\&c\le a,b\text { and }a\le b^c, \\&c\le a,b\text { and }a\le b\rightarrow c. \end{aligned}$$

Now assume $a\ge b$. Then $a\rightarrow b=a^b$ and

$$\begin{aligned}&\big (a\odot (a\rightarrow b)\big )\cap [b,1]\\&\quad =\big ({{\,\mathrm{Max}\,}}L(a,a^b)\big )\cap [b,1]\subseteq L(a,a^b)\cap [b,1]=\{b\}. \end{aligned}$$

On the other hand, $b\in L(a,a^b)$ and if $b\le c\in L(a,a^b)$ then $c\in L(a,a^b)\cap [b,1]=\{b\}$, i.e. $c=b$. This shows that $b\in {{\,\mathrm{Max}\,}}L(a,a^b)$ and hence $b\in \big ({{\,\mathrm{Max}\,}}L(a,a^b)\big )\cap [b,1]$, thus

$$\begin{aligned} \big ({{\,\mathrm{Max}\,}}L(a,a^b)\big )\cap [b,1]=\{b\}. \end{aligned}$$

proving divisibility of $(P,\le ,\odot ,\rightarrow ,1)$. $\square$

The divisibility has an essential influence on the logic for which the considered unsharply residuated poset is an algebraic semantics. Namely, if we know the truth values of *x* and $x\rightarrow y$ and we know that $y\le x$ then the truth value of *y* is exactly the conjunction of *x* and $x\rightarrow y$, which is just the derivation rule *Modus Ponens*.

If an unsharply residuated poset is a lattice, then clearly we have

$$\begin{aligned} x\odot y={{\,\mathrm{Max}\,}}L(x,y)={{\,\mathrm{Max}\,}}L(x\wedge y)=x\wedge y \end{aligned}$$

and the fact that $z\le x,y$ can be expressed by $x\vee z=x$ and $y\vee z=y$. Then unsharp adjointness can be formulated as follows:

$$\begin{aligned} (x\vee z)\odot (y\vee z)=z\text { if and only if }x\vee z\le (y\vee z)\rightarrow z. \end{aligned}$$

However, by (ii) of Proposition 2.4 we know that

$$\begin{aligned} (y\vee z)\rightarrow z=y\rightarrow z, \end{aligned}$$

and

$$\begin{aligned} (x\vee z)\odot (y\vee z)\ge z \end{aligned}$$

automatically holds. Hence the left-hand side of (iii) is equivalent to $(x\vee z)\odot (y\vee z)\le z$. Altogether, we obtain

$$\begin{aligned} (x\vee z)\odot (y\vee z)\le z\text { if and only if }x\vee z\le y\rightarrow z \end{aligned}$$

which is just relative adjointness as defined in Chajda et al. (2020) and mentioned above. This means that Definition 4.1 is compatible with the corresponding definition for lattices.

### Example 4.3

Let us consider the poset from Example 2.8. The table for $\odot$ looks as follows:

$$\begin{aligned} \begin{array}{c|c@{\quad }c@{\quad }c@{\quad }c@{\quad }c@{\quad }c@{\quad }c} \odot &{} 0 &{} a &{} b &{} c &{} d &{} e &{} 1 \\ \hline 0 &{} 0 &{} 0 &{} 0 &{} 0 &{} 0 &{} 0 &{} 0 \\ a &{} 0 &{} a &{} a &{} 0 &{} a &{} a &{} a \\ b &{} 0 &{} a &{} b &{} 0 &{} b &{} b &{} b \\ c &{} 0 &{} 0 &{} 0 &{} c &{} c &{} c &{} c \\ d &{} 0 &{} a &{} b &{} c &{} d &{} \{b,c\} &{} d \\ e &{} 0 &{} a &{} b &{} c &{} \{b,c\} &{} e &{} e \\ 1 &{} 0 &{} a &{} b &{} c &{} d &{} e &{} 1. \end{array} \end{aligned}$$

We can see that $d\odot e=\{b,c\}$ is not a singleton, and $b\in d\odot e$ implies $b\le d,e$ and $d\le d=e\rightarrow b$; also, conversely, $c\le e,d$ and $e\le e=d\rightarrow c$ imply $c\in \{b,c\}=e\odot d$.

## Conclusion

We constructed a binary operator on a finite poset with pseudocomplemented sections which can serve as an unsharp implication. It satisfies important properties required for implication in various sorts of propositional logics. Moreover, a negation derived by means of this implication satisfies the properties of implication in intuitionistic logic, thus our poset with this unsharp implication can be recognized as an algebraic semantics of a general case of intuitionistic logic. This approach enables us to introduce an algebraic semantics of intuitionistic logic which need not be distributive. This is not possible when traditional methods using, e.g. Heyting algebras are applied. Moreover, an unsharp conjunction is introduced having similar properties as those satisfied by the connective conjunction in propositional calculus. This unsharp conjunction together with the mentioned unsharp implication forms an adjoint pair. It means that a certain version of the derivation rule Modus Ponens can be considered in this logic. Hence, the logic based on such a poset can be considered as a fairly general kind of substructural logic.

## Data Availability

Not applicable.

## References

[CR1] Balbes R (1973). On free pseudo-complemented and relatively pseudo-complemented semi-lattices. Fund Math.

[CR2] Birkhoff G (1979) Lattice theory. AMS, Providence, R. I. 0-8218-1025-1

[CR3] Chajda I (2003). An extension of relative pseudocomplementation to non-distributive lattices. Acta Sci Math (Szeged).

[CR4] Chajda I, Kühr J, Länger H (2020). Relatively residuated lattices and posets. Math Slovaca.

[CR5] Chajda I, Länger H, Paseka J (2020). Algebraic aspects of relatively pseudocomplemented posets. Order.

[CR6] Chajda I, Radeleczki S (2003). On varieties defined by pseudocomplemented nondistributive lattices. Publ Math Debrecen.

[CR7] Cīrulis J (2008). Implication in sectionally pseudocomplemented posets. Acta Sci Math (Szeged).

[CR8] Cīrulis J (2014). Quasi-orthomodular posets and weak BCK-algebras. Order.

[CR9] Cornejo ME, Fariñas del Cerro L, Medina J (2021). A logical characterization of multi-adjoint algebras. Fuzzy Sets Syst.

[CR10] Curry HB (1977). Foundations of mathematical logic.

[CR11] Figallo-Orellano A (2016). A short note on the free implication algebra over a poset. South Amer J Log.

[CR12] Finch PD (1970). Quantum logic as an implication algebra. Bull Austral Math Soc.

[CR13] Frink O (1962). Pseudo-complements in semi-lattices. Duke Math J.

[CR14] Iturrioz L (1977). Łukasiewicz and symmetrical Heyting algebras. Z Math Logik Grundlagen Math.

[CR15] Köhler P (1980). A subdirectly irreducible double Heyting algebra which is not simple. Algebra Universalis.

[CR16] Köhler P (1981). Brouwerian semilattices. Trans Amer Math Soc.

[CR17] Liu H (2020). Pseudo-equality algebras and residuated posets. J Mult-Val Log Soft Comput.

[CR18] Nemitz WC (1965). Implicative semi-lattices. Trans Amer Math Soc.

[CR19] Nimbhorkar SK and Nehete JY, $$\delta $$-Ideals in pseudo-complemented distributive join-semilattices. Asian-Eur J Math **14** (2021), 2150106, 7 pp

[CR20] Ojeda-Hernández M, Cabrera I. P and Cordero P, Quasi-closed elements in fuzzy posets. J Comput Appl Math **404** (2022), Paper No. 113390, 9pp

[CR21] Rao MS (2012). $$\delta $$-ideals in pseudo-complemented distributive lattices. Arch Math (Brno).

[CR22] Talukder MR, Chakraborty HS, Begum SN (2021). $$\delta $$-ideals of pseudocomplemented semilattice. Afr Mat.

[CR23] Venkatanarasimhan PV (1971). Pseudo-complements in posets. Proc Amer Math Soc.

[CR24] Zeman JJ (1979). Normal implications, bounded posets, and the existence of meets. Notre Dame J Formal Logic.

